# BAC cloning and heterologous expression of a giant biosynthetic gene cluster encoding antifungal neotetrafibricin in *streptomyces rubrisoli*

**DOI:** 10.3389/fbioe.2022.964765

**Published:** 2022-08-15

**Authors:** Heung-Soon Park, Ji-Hee Park, Hye-Jin Kim, Seung-Hoon Kang, Si-Sun Choi, Eung-Soo Kim

**Affiliations:** Department of Biological Engineering, Inha University, Incheon, South Korea

**Keywords:** *streptomyces*, antifungals, antibiotics, genome mining, heterologous expression

## Abstract

Polyene natural products including nystatin A1, amphotericin B, ECO-02301, and mediomycin belong to a large family of valuable antifungal polyketide compounds typically produced by soil actinomycetes. A previous study (Park et al., Front. Bioeng. Biotechnol., 2021, 9, 692340) isolated *Streptomyces rubrisoli* Inha501 with strong antifungal activity and analyzed a large-sized biosynthetic gene cluster (BGC) of a linear polyene compound named Inha-neotetrafibricin (I-NTF) using whole genome sequencing and bioinformatics. In the present study, an entire I-NTF BGC (∼167 kb) was isolated through construction and screening of *Streptomyces* BAC library. Overexpression of the cloned I-NTF BGC in the wild-type *S. rubrisoli* Inha501 and its heterologous expression in *S. lividans* led to 2.6-fold and 2.8-fold increase in I-NTF yields, respectively. The qRT-PCR confirmed that the transcription levels of I-NTF BGC were significantly increased in both homologous and heterologous hosts containing the BAC integration of I-NTF BGC. In addition, the I-NTF aglycone-producing strains were constructed by a target-specific deletion of glycosyltransferase gene present in I-NTF BGC. A comparison of the *in vitro* biological activities of I-NTF and I-NTF aglycone confirmed that the rhamnose sugar motif of I-NTF plays a critical role in both antifungal and antibacterial activities. These results suggest that the *Streptomyces* BAC cloning of a large-sized natural product BGC is a valuable approach for natural product titer improvement and biological activity screening of natural product in actinomycetes.

## Introduction

Actinomycetes, with most being *streptomyces* species, are filamentous soil microorganisms with a high GC content and are widely known for producing highly valuable bioactive compounds (Bu et al., 2019). Thanks to these intrinsic merits, these species have been studied extensively to discover natural products useful for humans, such as medicinal or agricultural purposes, or to develop better derivatives (Bu et al., 2019). From a pharmaceutical viewpoint, actinomycetes-derived natural products provide important anticancer, antibiotic, anti-inflammatory, antiviral, antiparasitic, and antioxidant drugs (Manivasagan et al., 2014; Jakubiec-Krzesniak et al., 2018; Bu et al., 2019). In addition, with the increasing demand for novel anti-infectives to cope with growing drug resistance pathogens, various drug development strategies are being made *via* discovery and re-design of novel and cryptic BGCs in actinomycetes (Laxminarayan et al., 2013; Hobson et al., 2021). From an agricultural perspective, the recent development of eco-friendly pesticides using actinomycetes to prevent environmental pollution caused by indiscriminate use of chemical pesticides has been welcomed (Ab Rahman et al., 2018). In addition, its use is increasing because of the potential to promote growth and prevent pests and diseases through interactions with plants in the soil environment. (Ab Rahman et al., 2018; Shi et al., 2018; Kim et al., 2019). Nevertheless, novel natural product (NP) screening from the actinomycetes isolates is not that straightforward due to difficulty of cultivation of some wild isolates in laboratory conditions as well as limited production of potential metabolites. (Libis et al., 2019; Wang et al., 2021). In addition, the isolated metabolites are more likely to be re-isolation of already reported natural products rather than novel.

**FIGURE 1:**
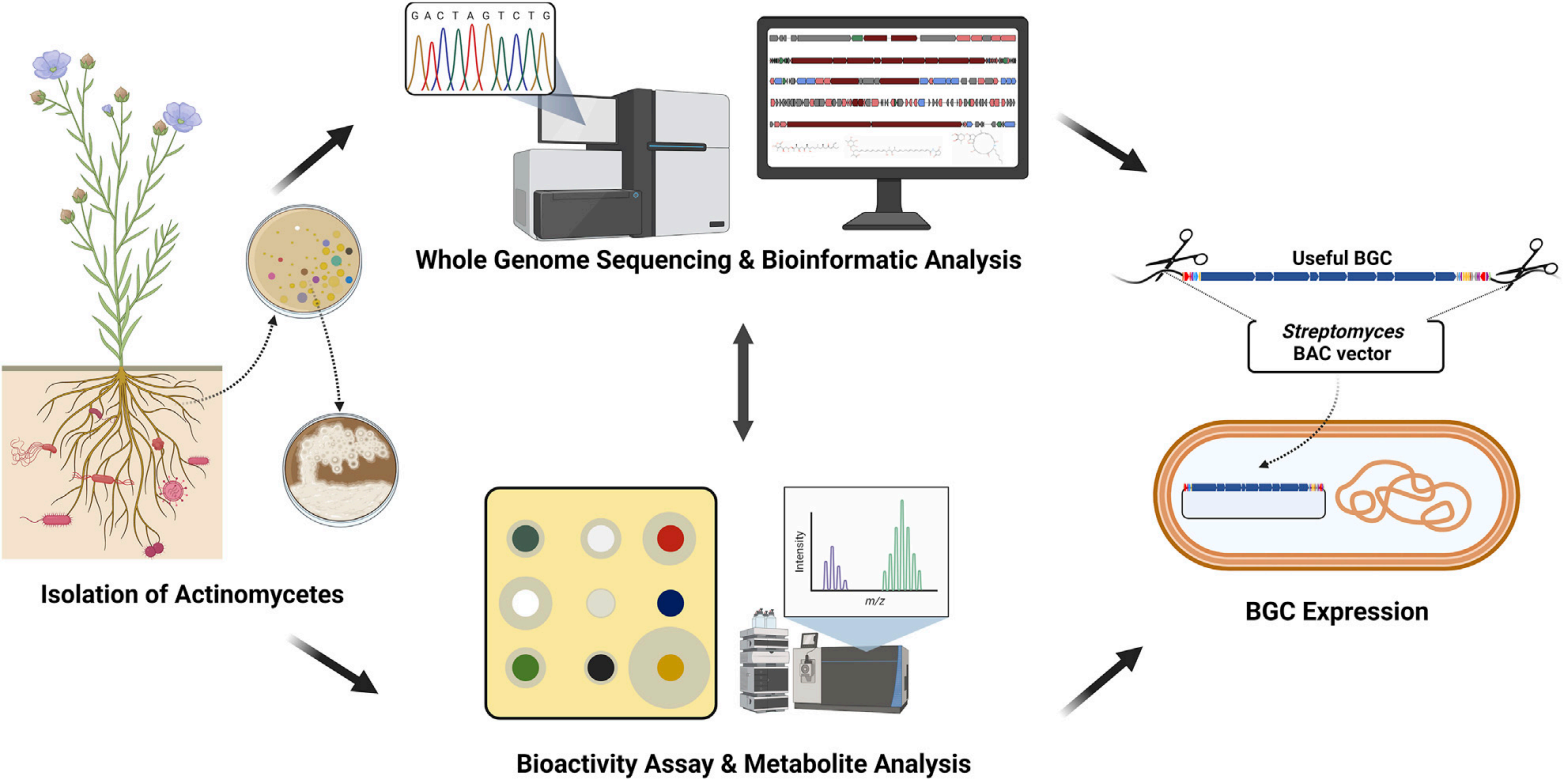
Actinomycetes-based genome mining scheme. First, actinomycetes were isolated from the soil. The isolated actinomycetes were analyzed for the whole genome sequence and metabolites. Finally, valuable biosynthetic gene clusters (BGCs) were isolated and expressed (Created with BioRender.com).

Thanks to genome mining and NGS, numerous NP BGCs derived from actinomycetes have been identified, and efforts to discover various biological activities using them are being pursued rapidly (Figure 1, Wang et al., 2021). As stated above, however, NP BGC expression in wild-type strains is mostly silent or very insignificant, so a target NP titer improvement strategy has to be developed (Nah et al., 2017; Choi et al., 2018; Kang and Kim, 2021). Currently, NP BGC cloning and heterologous expression are being attempted as the most popular strategies, among which TAR (transformation-associated recombination), CATCH (Cas9-assisted targeting of chromosome segments), and SBAC (*Streptomyces* bacterial artificial chromosome) methods were successfully practiced (Jiang et al., 2015; Nah et al., 2015; Kouprina and Larionov, 2016; Pyeon et al., 2017; Choi et al., 2019). Several *E. coli-Streptomyces* shuttle BAC vectors have been developed to carry the large-sized NP BGCs such as pStreptoBAC V, pSBAC, pESAC (Miao et al., 2005; Liu et al., 2009; Jones et al., 2013). The application of *E. coli-Streptomyces* BAC shuttle vector was successfully established through the precise cloning and heterologous expression of the type I polyketide (PK) BGCs of tautomycetin and pikromycin as well as the PK-nonribosomal peptide (NRP) hybrid BGC of meridamycin (Liu et al., 2009; Nah et al., 2015; Pyeon et al., 2017). Unique restriction enzyme recognition sites, either naturally existing or artificially inserted into both flanking regions, of the entire BGC were employed for capturing the BGCs.

Among the secondary metabolites produced by actinomycetes, polyenes, which exhibit antifungal activity, typically consist of a polyketide core with 20–40 carbon atoms, including 3–8 conjugated double bonds. The most well-known antifungal polyenes used primarily to treat fungal infections are polyketide macrolides, such as the tetraene-containing nystatin A1 and the heptaene-containing amphotericin B (Caffrey et al., 2016; Zhang et al., 2017). In addition to these typical macrocyclic polyene compounds, linear aminopolyol polyene compounds containing amino or guanidino moieties, such as linearmycin, ECO-02301, mediomycin, and neotetrafibricin are also reported to contain antifungal and antibacterial activity (Caffrey et al., 2016; Zhang et al., 2017). The polyene core is biosynthesized by a giant enzyme complex called polyketide synthase (PKS), followed by further post-PKS modification of the polyene core by tailoring enzymes, including P450 hydroxylases, sulfonyl transferase, and glycosyltransferases (Neumann et al., 2016).

In this study, I-NTF BGC was isolated through the construction and screening of the Inha501 BAC library (insert size of 200 kb), and the biosynthetic pathway of I-NTF was verified by heterologous expression. In addition, using the isolated BGC, various over- or heterologous expression *streptomyces* hosts were constructed to secure mutants with high I-NTF and I-NTF aglycone production. In addition, the effect of the rhamnose motif of I-NTF on the antifungal and antibacterial activity was confirmed by comparing I-NTF and I-NTF aglycone.

## Materials and methods

### Strains and growth conditions

*S. rubrisoli* Inha501 was distributed from Industrial Biomaterial Research Center, Korea Research Institute of Bioscience and Biotechnology (KRIBB), South Korea. *S. rubrisoli* Inha501 was grown routinely in ISP2 agar (malt extract 10 g, yeast extract 4 g, glucose 4 g, and agar 20 g per liter) at 30°C for the sporulation and seed culture, and *S. lividans* TK21 and *S. coelicolor* M511 were grown routinely in MS agar (soybean flour 20 g, D-mannitol 20 g, and agar 20 g per liter) at 30°C for the sporulation and seed culture. The transconjugants were grown on mISP4 (Difco™ ISP4 37 g, yeast extract 0.5 g, and Tryptone 1.5 g per liter) at 30°C. The R5 medium (sucrose 51.5 g yeast extract 2.5 g, peptone 5 g, malt extract 3 g, glucose 10 g, and 10N NaOH 0.7 ml per liter) was used to produce the I-NTF (Inha-neotetrafibricin A) and I-NTF aglycone. All *E. coli* strains were incubated at 37°C in Luria–Bertani medium supplemented with the appropriate antibiotics where needed. *Candida albicans* ATCC 14053, *Aspergillus niger* ATCC 9642, *Fusarium oxysporum* f. sp. *lactucae* KACC (Korean Agricultural Culture Collection) 42795, *Fusarium oxysporum* f. sp. *gladioli* KACC 40051, *Fusarium solani* KACC 44891, *Fusarium graminearum* KACC 47495, *Fusarium verticillioides* KCTC (Korean Collection for Type Cultures) 6065, *Fusarium semitectum* KCTC 16672, *Botrytis cinerea* KACC 40574*, Colletotrichum gloeosporioides* KACC 40003, *Curvularia lunata* KACC 40861, and *Alternaria alternata* KACC 40019 were grown on PDA medium (potato starch 4 g, glucose 2 g, agar 15 g per liter) at 28°C for 3 days.

### Bacterial artificial chromosome library construction of *S. rubrisoli* Inha501 and isolation of the entire I-NTF biosynthetic gene cluster into pSBAC-13

pESAC-13-Apramycin (*Bam*HI) was used for BAC library construction. The vector DNA was digested with *Bam*HI, dephosphorylated, and high-molecular-weight (HMW) DNA preparation from *S. rubrisoli* Inha501 was purified according to the standard procedure (Birren et al., 1997). A 1 ml of the culture solution was embedded in 2 ml of 2% (w/v) low-melting-point agarose plugs. The plugs were then treated with proteinase K at 50°C and stored in 0.5 M EDTA at 4°C. Partial digestion of the plugs was performed using five units of *Bam*HI per plug, and fixing the digestion time to 10–20 min at 37°C. The reactions were quenched by adding a 1/10 volume of 0.5 M EDTA (pH 8.0) on ice. Partially digested HMW DNA was size-selected on 1% (w/v) pulsed field agarose gels in 0.5X TBE on a CHEF DRIII (Bio-Rad, Canada). Two size selections were performed to increase the average insert size of the PACs. The first PFGE size selection was performed for 12 h at 14°C with ramped pulse times of 1–40 s and 6 V/cm, followed by a second size selection for 12 h at 14°C with ramped pulse times of 1–10 s and 4 V/cm. The DNA fragments, approximately 200 kb, were eluted from the gel by PFGE for 3 h with a constant pulse time of 30 s and 6 V/cm. The eluted DNA fragments were dialyzed against 1X TE (10 mM Tris-HCl, 1 mM EDTA, and pH 8.0) buffer for at least 2 h before ligation. The partially digested size-selected DNA fragments (80–100 ng) were ligated to 20 ng vector DNA in a volume of 50 μL with 1X ligase buffer and three units of ligase (USB, Canada) at 14°C for overnight incubation. A total volume of 100 μL of ligation was prepared. The ligation mixture was used to transform the *E. coli* DH10B (Invitrogen, United States) by electroporation. The cells were then selected on LB medium supplemented with 5% Sucrose plus apramycin by incubation at 37°C overnight. One thousand nine hundred and twenty clones with an average insert size of 200 kb were obtained from the library and screened by PCR using the I-NTF check primers in I-NTF BGC to identify pI-NTF (Supplementary Figure S1).

### Inactivation of I-NTF H gene (BAC modification) and I-NTF and I-NTF aglycone production strains construction

The I-NTF H gene in pI-NTF was deleted by inserting, the kanamycin-resistant gene into a flanking region of the I-NTF biosynthetic gene cluster using a PCR-targeted gene disruption system (Gust et al., 2003). The pI-NTF and pI-NTF*△i-ntf h* were introduced into the *Streptomyces* hosts (*S. rubrisoli* Inha501, *S. lividans* TK21, *S. coelicolor* M511) by triparental conjugation (Kieser et al., 2000). The cells from *E. coli* ET12567/pUB307 and *E. coli* DH10B/pI-NTF or *E. coli* DH10B/pI-NTF*△i-ntf* were collected at an OD_600nm_ of 0.4–0.6, washed twice with LB to remove the antibiotics, and then resuspended in 100 µL of LB. Then, the cells were mixed with 1 ml each of freshly activated spores of *S. rubrisoli* Inha501, *S. lividans* TK21, and *S. coelicolor* M511. The mixtures were plated on mISP4 and overlaid after approximately 16 h with the appropriate antibiotics. After 5 days of incubation, 10 colonies from each *Streptomyces* heterologous host were picked and streaked on MS or ISP2 plates containing the appropriate antibiotics. The insertion of pI-NTF or pI-NTF*△i-ntf* into the *Streptomyces* hosts’ chromosomes was checked by PCR (Supplementary Figures S3, S6).

### Production and purification of I-NTF and I-NTF aglycone

I-NTF and I-NTF aglycone production strains were inoculated in 100 ml of TSB medium at 30°C and 220 rpm for 2 days. The pre-cultures were added to 2 L of R5 medium in a 5 L bioreactor for batch fermentation. After 7 days of cultivation, the culture broth was extracted in 2 L of *n*-butanol. The extract was concentrated using a vacuum evaporator. The concentrated extract was dissolved in methanol and loaded onto a column packed with a C18 reversed-phase silica gel (Daiso, Japan) and eluted with methanol-water (30:70, v/v) to remove any residual sugar from the production media. The extracts with the sugar removed were purified using a fraction collector (Interchim, France) on a gradient comprised of solvents A (water) and B (methanol): 30% B (v/v) (0–10 min) and 100% B (v/v) (100 min) at a flow rate of 20 ml/min. The fractions containing I-NTF or I-NTF aglycone with >90% purity were detected at 332 nm and analyzed by high-performance liquid chromatography (HPLC). The column was equilibrated with 60% solvent A (0.05 M ammonium acetate, pH 6.5) and 40% solvent B (acetonitrile). The flow rate was set to 0.5 ml/min under the following conditions: 0–30 min and 40% B.

### LC-MS/MS analysis

The polyene compounds showing >90% purity were analyzed by Waters H-Class Acquity system coupled to Quadruple Time-of -Flight (Q-ToF) from Waters. MS was conducted in positive ion modes over a mass range from *m*/*z* 50 to *m*/*z* 2500 using an electrospray ionization source. The capillary voltage was 3.0 kV and source temperature was 100°C. Sampling cone and source offset were 40 V and 80 V, respectively. Desolvation temperature and gas flow were 250°C and 600 L/h. For the chromatographic conditions, solutions A (0.1% formic acid in distilled water) and B (0.1% formic acid in acetonitrile) were used for elution and loaded onto a Phenomenex Kinetex 1.7 m C18 (2.1 mm × 150 mm, 1.7 mm). The flow rate was set to 0.4 ml/min under the following conditions: 0–1 min, 90% A; 1–5 min, 90%–50% A; 5–18 min, 50%–0% A; 18–25 min, 0% A; 25–27 min, 0%–90% A; and 27–30 min, 90% A.

### RNA analysis by qRT-PCR

RNA was prepared using a RNeasy Mini Kit [Qiagen, Germany]. cDNA conversion was carried out using a PrimeScript first strand cDNA Synthesis Kit (TaKaRa, Japan) according to the manufacturer’s instructions. Real-time RT-PCR was performed using TaKaRa SYBR Premix Ex Taq (Perfect Real Time) with a Thermal Cycler Dice Real Time System Single (code TP850) (TaKaRa, Japan). Supplementary Table S1 lists the primer pairs. The PCR conditions included activation for 10 min at 95°C, followed by 35 cycles of 30 s at 95°C, 30 s at 58°C, and 30 s at 72°C. The data were collected during each 72°C step, and melting curve analysis was performed at default settings ranging from 60°C to 95°C. The relative level of amplified mRNA was normalized to the mRNA expression level of the housekeeping gene, *Streptomyces hrdB*, which was amplified as an internal control using the primer pair *hrdB*_F (5′- GCGGTGGAGA AGTTCGACTA -3′) and *hrdB*_R (5′- TTG​ATG​ACC​TCG​ACC​ATG​TG -3′) (Han et al., 2019).

### Antibacterial assay of I-NTF and I-NTF aglycone

I-NTF and I-NTF aglycone were assessed using the paper disc diffusion method. The bioassay was performed using *Staphylococcus aureus*, *Bacillus subtilis*, *E. coli*, and *Corynebacterium glutamicum* as the indicator organism. Samples (1 ml) of overnight-cultured bacteria were mixed with 4 ml of a sterile solution of 0.5% agarose in H_2_O. The mixtures were spread on prewarmed NA (peptone 5 g, beef extract 3 g, and agar 15 g per liter), and then solidified for 30 min. The discs (6 mm diameter) were then loaded with 10 μg of the extracts (dissolved in methanol). After incubation for 24 h, the inhibition zone surrounding the discs, resulting from the diffusion of I-NTF and derivatives, was observed.

### Antifungal assay of I-NTF and I-NTF aglycone

The Clinical and Laboratory Standards Institute document M27-A3 was adapted to the *in vitro* antifungal assay (Rex et al., 2008). After the fungus was cultured in PDB medium at 30°C for 3 days, the cultured solution was diluted with PDB medium until the OD value reached 0.3 at 530 nm. A working suspension was prepared by a 1:2000 dilution with RPMI-1640 broth media (with glutamine and phenol red, without bicarbonate, Sigma-Aldrich, United States), which resulted in 5.0 × 10^2^–2.5 × 10^3^ cells per μl. 10 μL of the DMSO containing polyene antibiotics at various concentrations (25–1,600 μg/ml) were added to the working suspension of 990 μL, and the mixtures were then incubated at 30°C without shaking for 3 days. The colorimetric change in the mixture from red to yellow indicated the growth of the fungus*.* The minimum inhibitory concentration (MIC) was determined by measuring the minimum concentration that changed the color to yellow. The experiment was performed in duplicate.

## Results

### Bacterial artificial chromosome library construction of *S. rubrisoli* Inha501 and isolation of the I-NTF biosynthetic gene cluster

**FIGURE 2:**
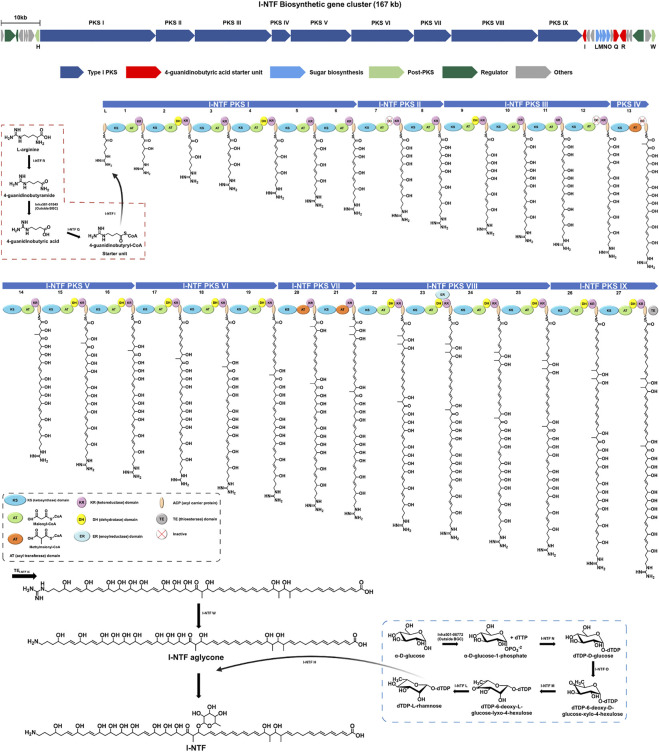
Proposed biosynthetic pathway of I-NTF from *S. rubrisoli* Inha501. The red dash line is the start unit (4-guanidinobutyric acid) synthesis, and the blue dash line is the sugar motif (rhamnose) synthesis.

**FIGURE 3:**
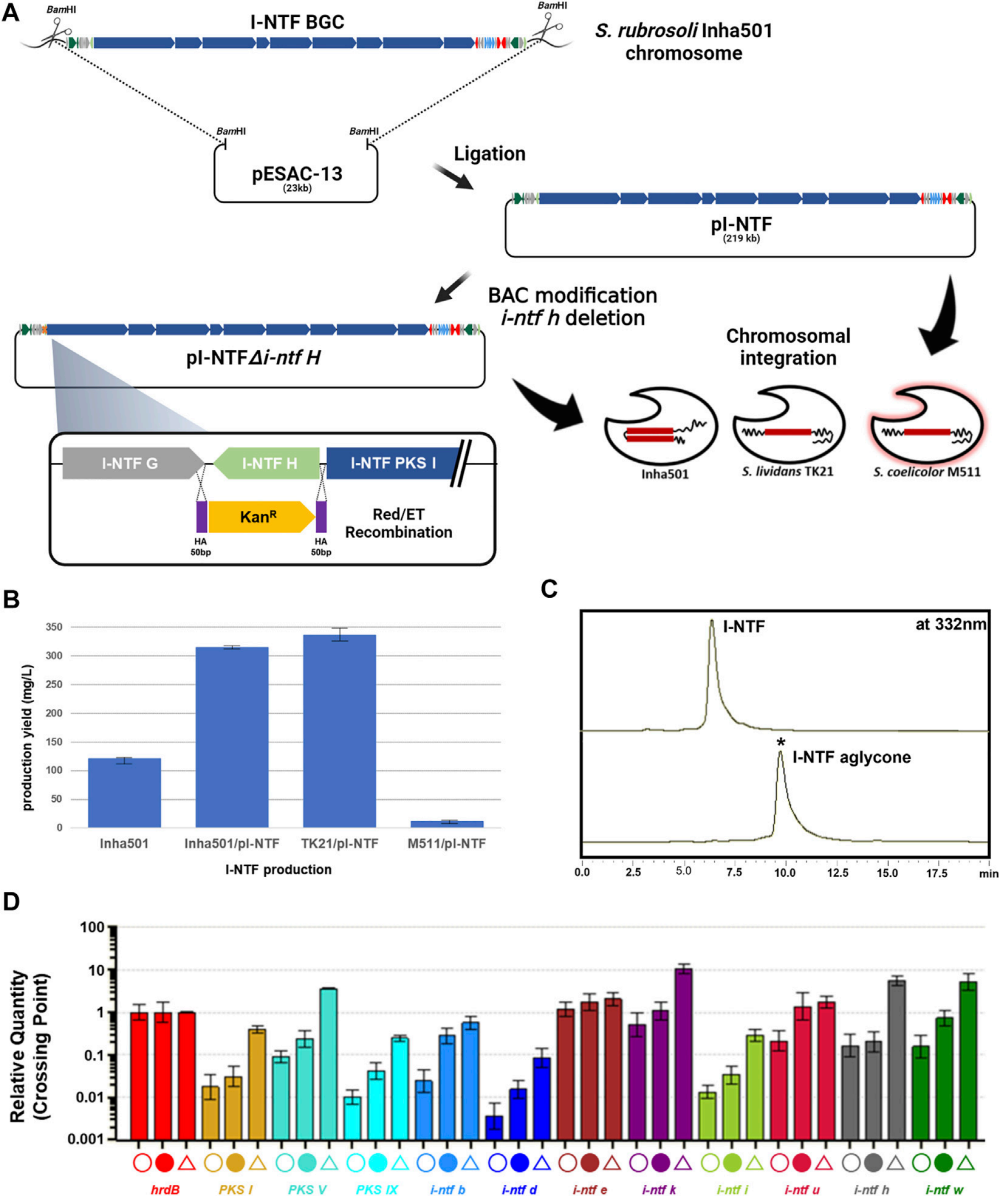
**(A)** Construction scheme of I-NTF and I-NTF aglycone production strains. **(B)** Comparison of I-NTF production yields after 7 days of culture. **(C)** HPLC chromatograms of the cultures from the *S. rubrisoli* Inha501 (upper line) and the *i-ntf h* disrupted *S. rubrisoli* Inha501 mutant (lower line). The asterisked peak is confirmed to be the I-NTF aglycone. **(D)** Transcript analysis of I-NTF production strains by qRT-PCR. Open circle, transcripts from *S. rubrisoli* Inha501 at 120 h; closed circle, transcripts from the I-NTF over-production mutant (*S. rubrisoli* Inha501/pI-NTF) at 120 h; open triangle, transcripts from the I-NTF heterologous production mutant (*S. lividans* TK21/pI-NTF) at 120 h; house-keeping gene, hrdB (red); PKS genes, PKS I (yellow), PKS V (emerald), and PKS IX (sky blue); regulatory genes, I-NTF B (blue) and I-NTF U (dark red); Post-PKS genes, I-NTF H (gray) and I-NTF W (dark green); other genes, I-NTF D (dark blue), I-NTF E (brown), I-NTF K (dark purple), and I-NTF I (green). All transcript measurements were performed in duplicate (Created with BioRender.com).

A previous study, confirmed that I-NTF with antifungal activity was produced by #22 BGC, containing nine type I PKS genes, in *S. rubrisoli* Inha501 (Park et al., 2021, Figure 2). As shown in Figure 2, a total of 27 modules in nine PKS genes is proposed to generate a linear amino polyol polyketide backbone, followed by guanidine removal and rhamnose glycosylation by I-NTF W and I-NTF H, respectively (Zhang et al., 2017; Park et al., 2021). A BAC library of 200 kb insert size of *S. rubrisoli* Inha501 was constructed to isolate 167 kb I-NTF BGC (Figure 3A). The total DNA of *S. rubrisoli* Inha501 was purified and digested partially with *Bam*HI to summarize the BAC library construction. Approximately 200 kb of the DNA fragments were then purified by CHEF. Finally, the purified DNA fragments were cloned into the *E. coli* - *Streptomyces* shuttle BAC vector (pESAC-13) and screened by antibiotics and PCR (Supplementary Figure S1; Supplementary Table S1). Two positive colonies containing complete I-NTF BGC were obtained; the insert size was approximately 200 kb through CHEF (Supplementary Figure S2). One colony was selected, and it was confirmed that complete I-NTF BGC was included by PCR (Supplementary Figure S3). As a result, I-NTF BGC was isolated successfully. The selected BAC vector was called pI-NTF (∼200 kb), and the vector was used to confirm the biosynthetic pathway of I-NTF.

### Homologous or heterologous expression of the I-NTF biosyntheticgene cluster

The I-NTF BGC was overexpressed by introducing pI-NTF in *S. rubrisoli* Inha501 to determine the I-NTF production titer (Figure 3A). When introduced into the wild type, the pI-NTF derived from pESAC-13, a *Φ*C31-based vector, can be integrated into the attB site or homologous recombination into the #22 BGC in *S. rubrisoli* Inha501. Five ex-conjugates were confirmed by attP check PCR and all conjugates were confirmed to be homologous recombination (Supplementary Figure S4). This is believed that the efficiency of homologous recombination was higher than that of the integration to attB in *S. rubrisoli* Inha501, as the homologous arm was approximately 200 kb. As a result, the level of I-NTF production was increased from 121 mg/L to 315 mg/L (approximately 2.6-fold) (Figure 3B).

pI-NTF was next introduced into *S. lividans* TK21 and *S. coelicolor* M511 to determine whether the I-NTF was produced through heterologous expression. Unlike the wild type, it was integrated into the attB site, and I-NTF production was confirmed in the heterologous host (Figure 3B, Supplementary Figure S5). *S. coelicolor* M511/pI-NTF produced a relatively low level of I-NTF production (11 mg/L), while *S. lividans* TK21/pI-NTF increased production to 336 mg/L (approximately 2.7-fold) (Figure 3B). LC-MS analysis of the purified I-NTF (>90% purity) in the *S. lividans* TK21/pI-NTF culture revealed a signal at m/z 1226.73 (calculated mass, 1227.73), which is the same as the signal at m/z 1226.73 for [C_67_H_104_NO_19_]^-^ of I-NTF (Park et al., 2021, Supplementary Figure S5).

The transcription level of the I-NTF biosynthetic genes was analyzed to understand the molecular basis responsible for the enhanced I-NTF production in the over- or heterologous I-NTF overproducing strain. qRT-PCR analysis showed that the transcription of all genes in I-NTF BGC, including the PKS genes (*i-ntf PKS I*, *i-ntf PKS V,* and *i-ntf PKS IX*) and post-PKS genes (*i-ntf h* and *i-ntf w*), was increased in the I-NTF overproducing strain compared to the wild type (Figure 3D, Supplementary Table S1).

### Construction of the I-NTF aglycone production strain

In polyene, the sugar motif is closely related to biological activity, such as enhancing the antifungal activity or reducing toxicity (Kim et al., 2015; Kim et al., 2017). To confirm the role of the sugar motif (rhamnose) in I-NTF and identify the biosynthetic pathway of I-NTF, the I-NTF aglycone production strain was constructed through an *i-ntf h* deletion in pI-NTF (Figure 3A). The *i-ntf h* gene in pI-NTF was disrupted by a homologous recombination using the BAC modification system, and the mutation was verified genetically by PCR product sequencing analysis (Figure 3A, Supplementary Figure S6, Nah et al., 2015). The constructed BAC vector was called pI-NTF*△i-ntf h* and introduced into *S. rubrisoli* inha501, *S. lividans* TK21, and *S. coelicolor* M511 to determine if I-NTF aglycone was produced. In *S. rubrisoli* Inha501, *i-ntf h* was substituted for the kanamycin-resistant gene by a homologous recombination. In other strains, it was integrated into the attB site. HPLC analysis showed that, the polyene peak with a different retention time from I-NTF had been purified (Figure 3C). LC-MS of purified the polyene peak contained a signal at m/z 1082.66 for [C_61_H_95_NO_15_]^+^ (calculated mass of I-NTF aglycone is 1081.67), confirming that I-NTF aglycone had been putatively produced, as expected in the constructed mutant strain (Figure 3C, Supplementary Figure S7). Furthermore, I-NTF aglycone was produced at 30 mg/L of *S. rubrisoli* Inha501*△i-ntf h*, 12 mg/L of *S. lividans* TK21/pI-NTF*△i-ntf h*, and 160 mg/L of *S. coelicolor* M511/pI-NTF*△i-ntf h* (Supplementary Figure S8).

### Antifungal and antibacterial assays of I-NTF and I-NTF aglycone

**FIGURE 4:**
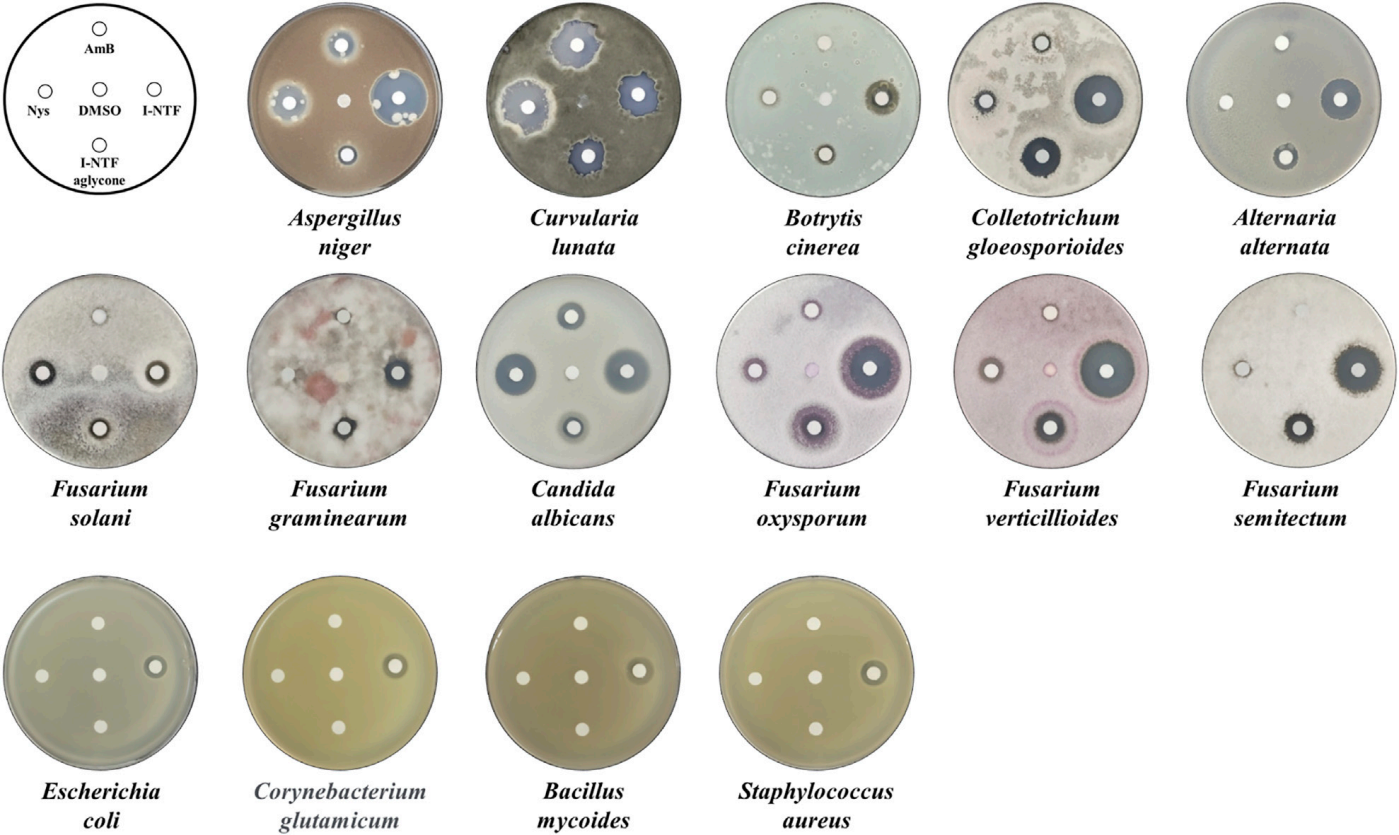
Antifungal and antibacterial assay. Comparison of the antifungal activity against 11 fungi and antibacterial activity against four bacteria. 10 μg of each polyene was loaded on a paper disc and placed on each plate. 3 days passed after being placed on the PDA plate containing fungus and 1 day passed after being placed on the NA plate medium containing bacteria. AmB, amphotericin B; Nys, nystatin A1; DMSO, dimethyl sulfoxide (Negative control).

The purified I-NTF and I-NTF aglycone were evaluated for their *in vitro* antifungal and antibacterial activities using the paper disc diffusion method and MIC (minimum inhibitory concentration) evaluation assays (Figure 4; Table 1). In the case of the antifungal assay using the paper disc diffusion method, the purified polyenes were performed in the assay against 11 fungi (*Aspergillus niger*, *Curvularia lunata*, *Botrytis cinerea*, *Colletotrichum gloeosporioides*, *Alternaria alternata*, *Candida albicans*, *Fusarium oxysporum*, *Fusarium verticilliodes*, *Fusarium semitectum*, *Fusarium solani*, and *Fusarium graminearum*). Although they exhibited antifungal activity against all the fungi tested, the inhibition zone of I-NTF was always larger than that of I-NTF aglycone (Figure 4). In addition, the purified polyenes were measured by examining the MIC evaluation assays of the purified I-NTF using the colorimetric change in the RPMI-1640 media containing the fungus (Supplementary Figure S10, Rex et al., 2008). In all fungi, the MIC value of I-NTF was lower than that of I-NTF aglycone (Table 1). Hence, the sugar motif of I-NTF plays a significant role in the antifungal activity.

**TABLE 1:** *In vitro* antifungal activity of polyene.

	Amphotericin B	Nystatin A	I-NTF	I-NTF aglycone
Antifungal activity (MIC^a^, μg/ml)				
*C. albicans* ATCC 14053	0.5	4	2	8
*A. niger* ATCC 9642	0.5	4	2	>16
*F. oxysporum* KACC 40051	2	8	2	8
*F. oxysporum* KACC 42795	1	8	4	8
*F. verticillioides* KCTC 6065	1	8	<0.25	2
*F. semitectum* KCTC 16672	1	4	0.5	2
*A. alternata* KACC 40019	>16	>16	4	8
*C. lunata* KACC 40861	16	>16	4	16
*C. gloeosporioides* KACC 40003	0.5	2	4	8
*B. cinerea* KACC 40574	1	2	16	>16

a Minimum inhibitory concentration (values resulting in no visible fungal growth).

In the case of the antibacterial assay using the paper disc diffusion method, the purified polyenes were evaluated in an antibacterial assay against four bacteria (*Staphylococcus aureus*, *Bacillus subtilis*, *E. coli*, and *Corynebacterium glutamicum*). Among the polyenes assessed, only I-NTF exhibited antibacterial activity (Figure 4). Interestingly, the sugar motif of I-NTF plays an important role in the antibacterial activity as well as antifungal activity.

## Discussion

Coning and heterologous expression of BGC has played a tremendous role in NP research with the advances in NGS technologies and bioinformatics that allow for the rapid and systematic identification of known and cryptic BGCs from many actinomycetes genome sequences. The innovations in synthetic biology have also facilitated the process of heterologous expression by providing tools for rapid cloning and engineering of NP BGCs to enhance production yield or to awaken cryptic BGCs (Kang and Kim, 2021). However, cloning and heterologous expression of an entire NP BGC, often as large as over 100 kb, still remain tricky due to the ineffectiveness of genetic systems in manipulating large-sized NP BGCs (Nah et al., 2017). The utility of *E. coli-Streptomyces* shuttle BAC vector system was successfully established through the precise cloning and heterologous expression of several large-sized BGC including tautomycetin BGC (∼80 kb), pikromycin BGC (∼60 kb), daptomycin BGC (∼128 kb), and meridamycin BGC (∼90 kb) (Nah et al., 2017; Pyeon et al., 2017).

Here, approximately 170 kb of the I-NTF BGC in *S. rubrisoli* Inha501 was successfully isolated by constructing a *E. coli-Streptomyces* shuttle BAC library with an insert size of 200 kb and the proposed biosynthetic pathway of I-NTF was verified through heterologous expression (Figures 2, 3). Although *S. lividans* TK21 host containing the pI-NTF exhibited increased production of I-NTF up to 336 mg/L, *S. coelicolor* M511 host containing the pI-NTF produced a relatively low level (11 mg/L) of I-NTF (Figure 3B). These results imply that the selection of ideal heterologous host is a very important factor that affects the success of the heterologous expression of NP BGCs. There might be an unknown gene(s) regulating I-NTF BGC negatively only in *S. coelicolor*, which needs to be further identified. Two copies of the I-NTF BGC in *S. rubrisoli* Inha501 *via* BAC-driven tandem repeat approach exhibited a 2.6-fold increase (∼316 mg/L) in I-NTF production by increased biosynthetic gene expression level, implying presences of multi-copies biosynthetic genes are very effective for homologous overexpression.

Various polyene compounds with a sugar motif by glycosyltransferase among several post-PKS modifications have been reported (Caffrey et al., 2022). Interestingly, the sugar moiety of polyene has proved to play an important role in maintaining the antifungal activity, reducing toxicity, or increasing solubility (Caffrey et al., 2022). This study evaluated the effect of sugar moiety (rhamnose) in I-NTF by deleting, *i-ntf h* (glycosyltransferase) in pI-NTF using the BAC modification method (Nah et al., 2015). Using this method, it was possible to obtain an I-NTF aglycone-producing *S. coelicolor* strain with 32 times higher (∼ 160 mg/L) production than the *S. rubrisoli* Inha501 I-NTF aglycone-producer. *In vitro* antifungal and antibacterial assays were then performed on I-NTF and I-NTF aglycone. As expected, the sugar motif of I-NTF played an essential role in its antifungal and antibacterial activities. Further studies need to be pursued to determine the correlation between the biological activities of polyene and the presence of sugar moiety. Although the bioassay of I-NTF aglycone didn’t showed any visible antibacterial activity, other studies on linearmycin, of which structure is quite similar with I-NTF aglycone, was reported to maintain antibacterial activity without sugar moiety (Figure 4, Nguyen et al., 2021). Further studies will follow to elucidate the antibacterial mechanism of I-NTF as well as the significance of the sugar moiety of I-NTF.

In summary, this paper describes the isolation of the giant I-NTF BGC present in the *S. rubrisoli* Inha501 strain, highlighting the proposed biosynthetic pathway through heterologous expression and confirming the *in vitro* biological activities of I-NTF and I-NTF aglycone. The heterologous expression of I-NTF BGC enhanced its production tier by 2.7-fold higher than that from the wild type, and the I-NTF aglycone-producing heterologous host strain produced approximately 32 times more than the I-NTF aglycone-producing *S. rubrisoli* Inha501 mutant strain. In the *in vitro* antifungal and antibacterial assay of I-NTF and I-NTF aglycone, some phytopathogenic fungi showed higher activity than Amphotericin B and Nystatin A1, and these results confirmed the potential application of I-NTF for biological agents. Moreover, the sugar moiety of I-NTF could play a critical role in controlling the antifungal and antibacterial activities. Overall, these results suggest that potentially valuable BGCs can be selected through genome mining of diverse actinomycetes, and their heterologous expressions in optimally engineered strains will continue to play a pivotal role in genomics-driven NP research and development.

## Data Availability

The raw data supporting the conclusion of this article will be made available by the authors, without undue reservation.
