# Supplementary material for: BAC cloning and heterologous expression of a giant biosynthetic gene cluster encoding antifungal neotetrafibricin in streptomyces rubrisoli
Source: Front Bioeng Biotechnol. 2022 Aug 15;10:964765. doi: 10.3389/fbioe.2022.964765 (PMC9421130; doi:10.3389/fbioe.2022.964765)
Supplement: Supplementary file 1 [file DataSheet1.docx]

**BAC cloning and heterologous expression of a giant biosynthetic gene cluster encoding antifungal neotetrafibricin in *Streptomyces rubrisoli***

**Heung-Soon Park, Ji-Hee Park, Hye-Jin Kim, Seung-Hoon Kang, Si-Sun Choi, and Eung-Soo Kim^*^**

Department of Biological Engineering, Inha University, Incheon 22212, Republic of Korea

^*^Corresponding author

E-mail: eungsoo@inha.ac.kr; Phone: +82-32-860-8318; Fax: +82-32-865-4046

Running title: *Streptomyces* BAC cloning and heterologous expression of neotetrafibricin BGC

Key-words: *Streptomyces*, antifungals, antibiotics, genome mining, heterologous expression

**Supplementary Figure S1.** Screening of the positive clones containing I-NTF BGC through PCR confirmation after BAC library construction of *Streptomyces rubrisoli* Inha501. 1; clone 1, 2; clone 2, 3; clone 3, 4; clone 4, CK4; positive control with genomic DNA 501-I-NTF up, CK5; positive control with genomic DNA 501-I-NTF middle, CK6; positive control with genomic DNA 501-I-NTF down, M; 200bp marker (Cat. M200-8R, Bio S&T).

**
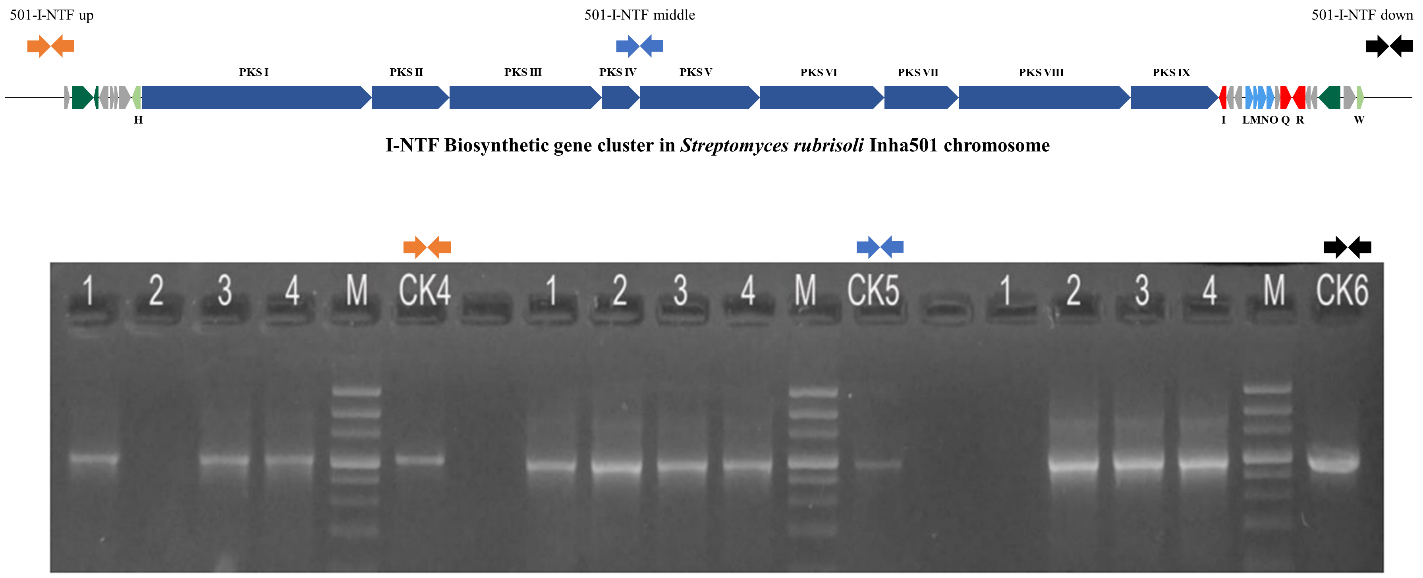
**

**Supplementary Figure S2.** Insert size determination of the selected positive clones containing I-NTF BGC by CHEF (Dra I digestion). 1; clone 1, 2; clone 2 (Including Dra I recognition site), 3; clone 3 (Including Dra I recognition site), 4; clone 4, M1; Lambda PFG ladder, M2; 0.2-10kb marker (Cat. S200-13R, Bio S&T).


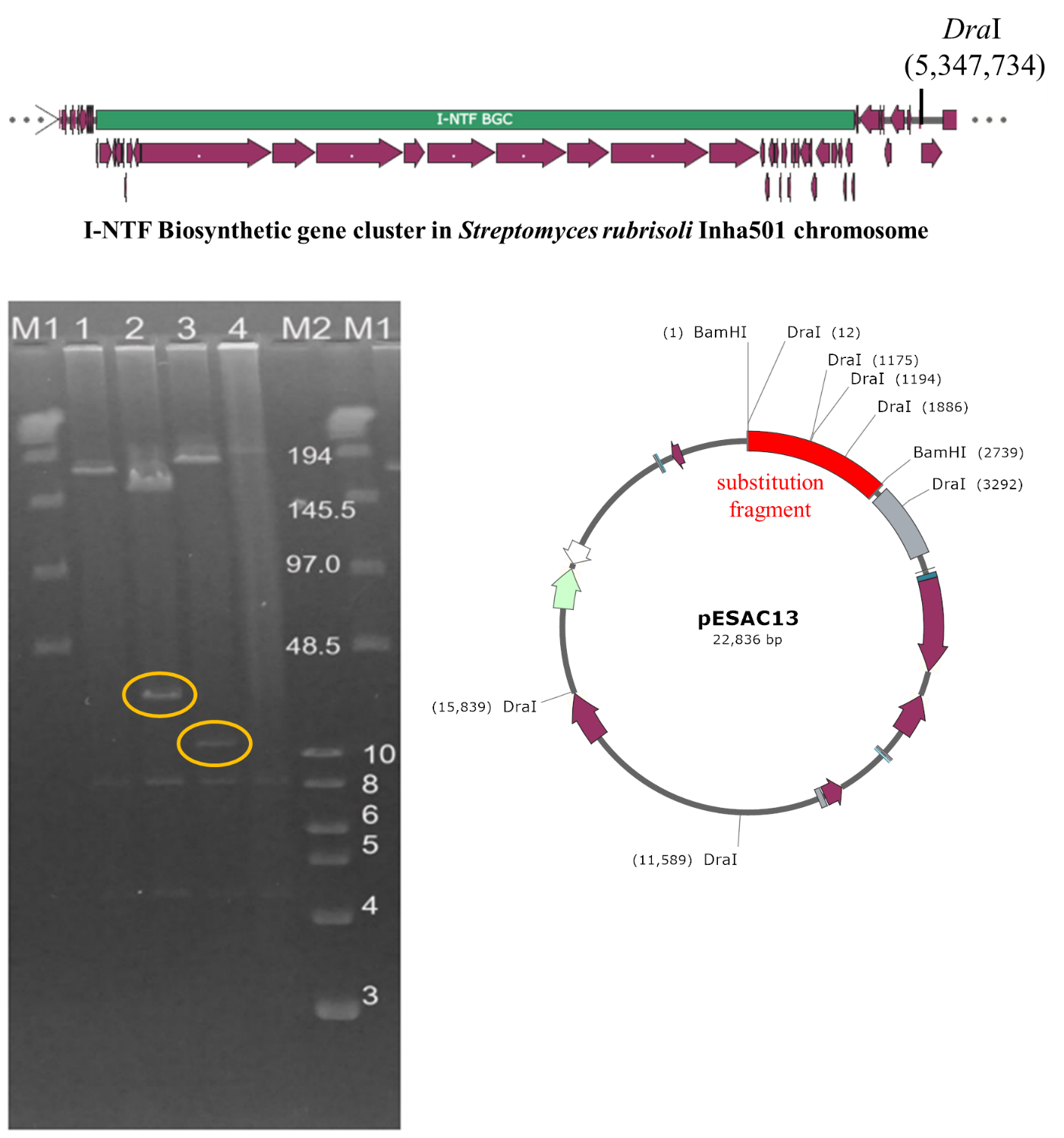


**Supplementary Figure S3.** Confirmation of the selected clone 3 by PCR using I-NTF check primer in I-NTF BGC. Supplementary Table S1 lists the primer pairs. M; 100bp ladder (Cat. BM301-01, TransGen Biotech).


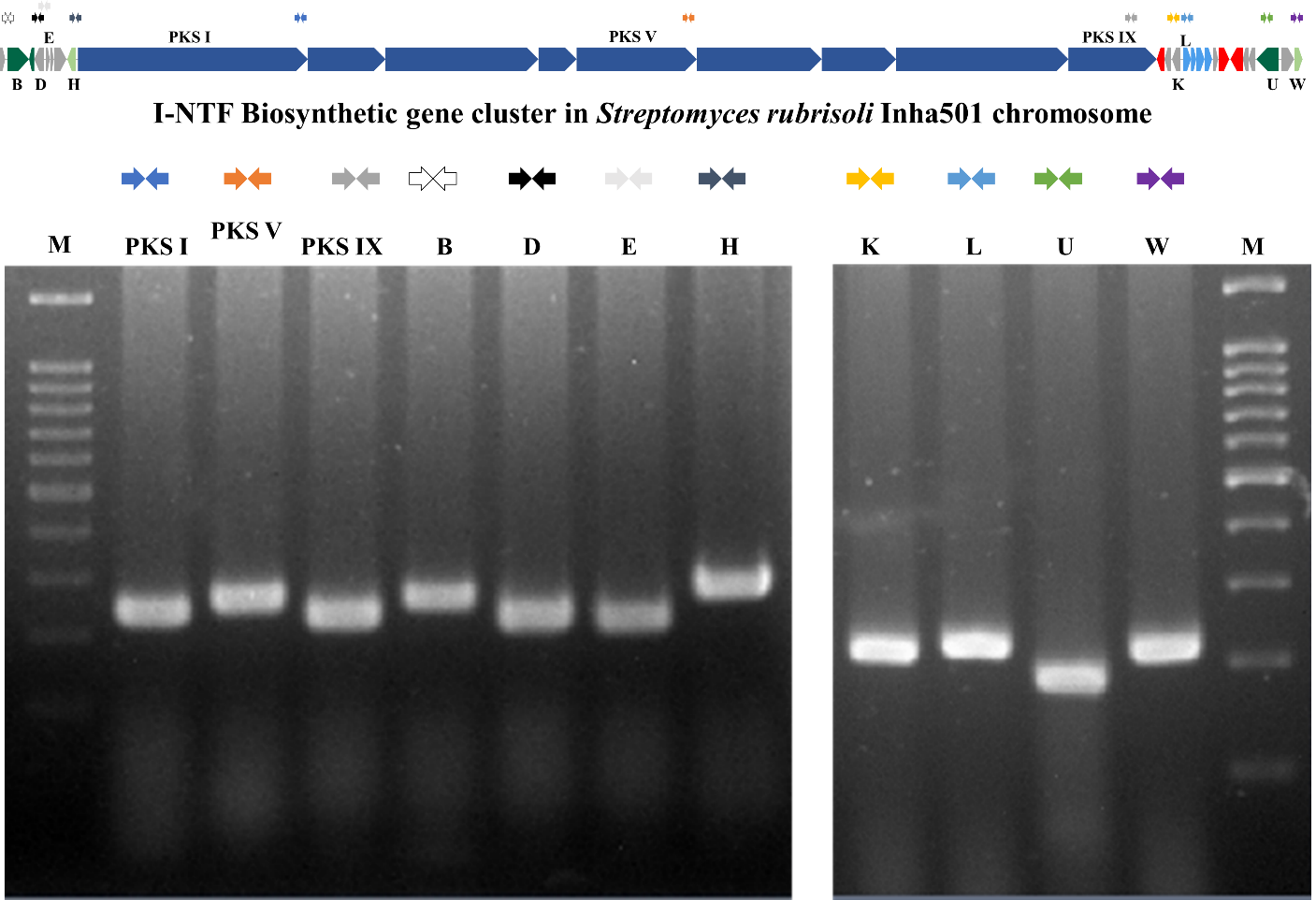


**Supplementary Figure S4.** Confirmation of the attp site in pESAC-13 (Check primer pair; attp_F (5’-GTACTGACGGACACACCGAA-3’) and attp_R (5’- GTCCCGAAGGATTCGCATAA-3’). M; 1kb plus DNA ladder (Cat. BM211-01, TransGen Biotech).

**
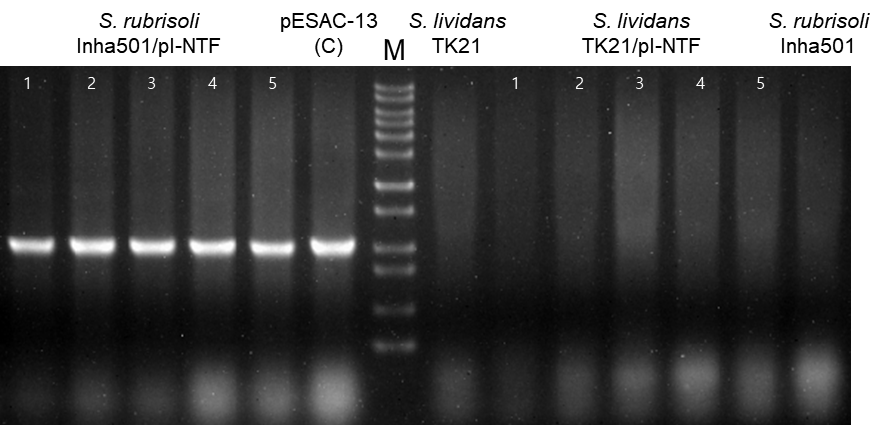
**

**
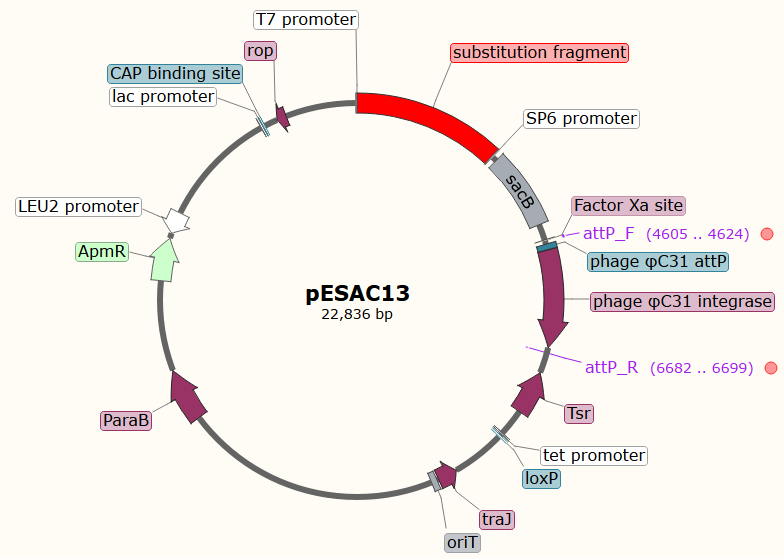
**

**Supplementary Figure S5.** Comparison of HPLC Analysis Results of *S. rubrisoli* Inha501 and *S. lividans* TK21/pI-NTF at 332nm. High Resolution Mass Spectrometry (HRMS) in positive ion mode to determine the production of I-NTF.


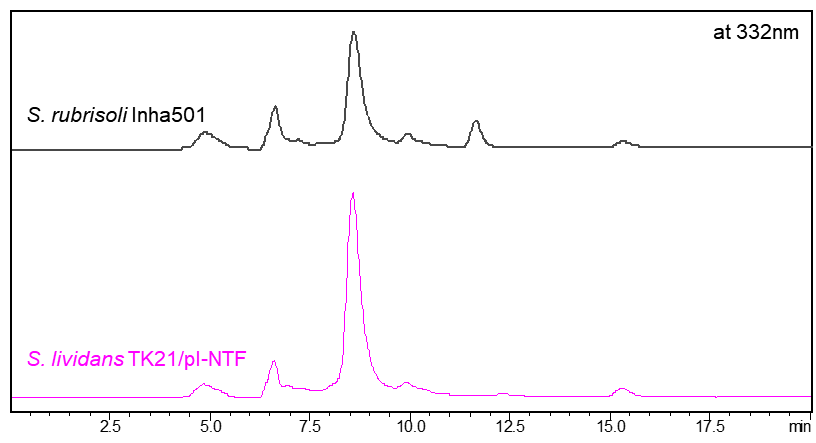


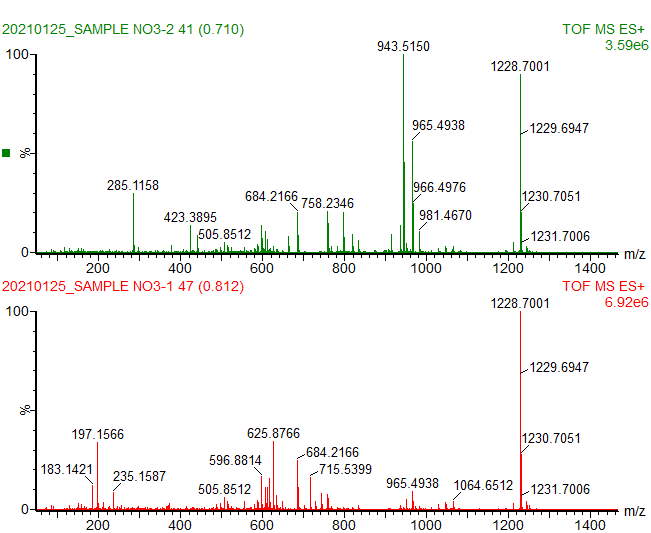


**Supplementary Figure S6.** Deletion of *i-ntf h* from pI-NTF. Genetic confirmation using the following primer pair: Check F (5’- TTCTTGCCGCCAAGGATCTG -3’) and Check R (5’- TCGCTTGGTCGGTCATTTCG -3’). M; 1kb plus DNA ladder (Cat. BM211-01, TransGen Biotech).

\
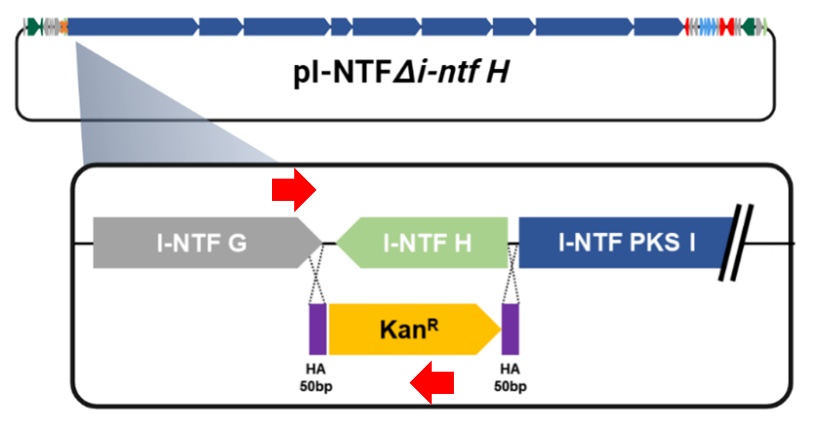


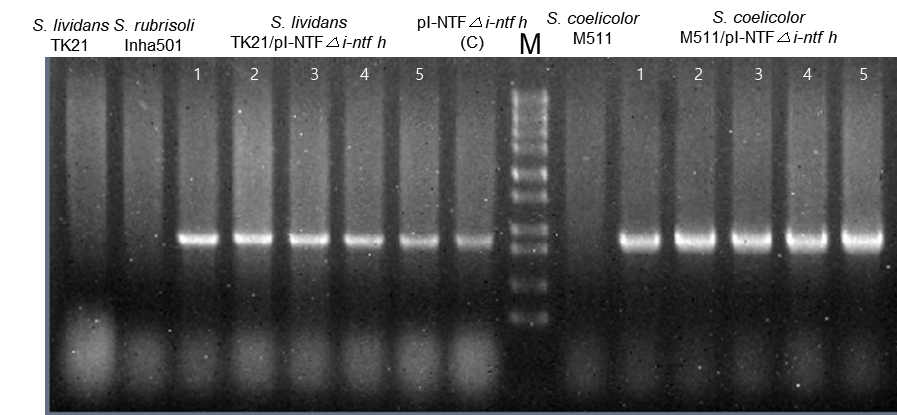


**Supplementary Figure S7.** Comparison of HPLC Analysis Results of *S. rubrisoli* Inha501 and *S. rubrisoli* Inha501/pI-NTF*△i-ntf h* at 332nm. High Resolution Mass Spectrometry (HRMS) in positive ion mode to determine the production of I-NTF aglycone.


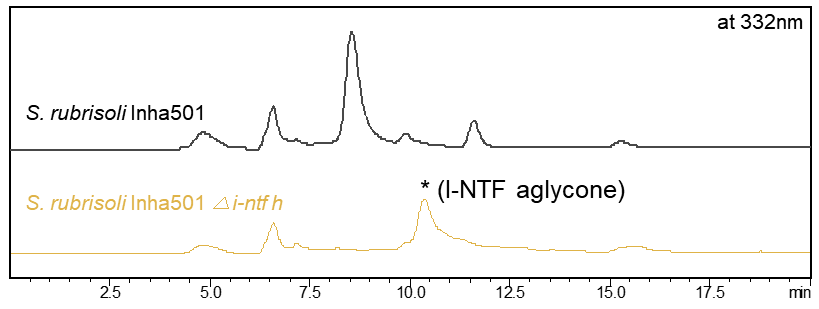


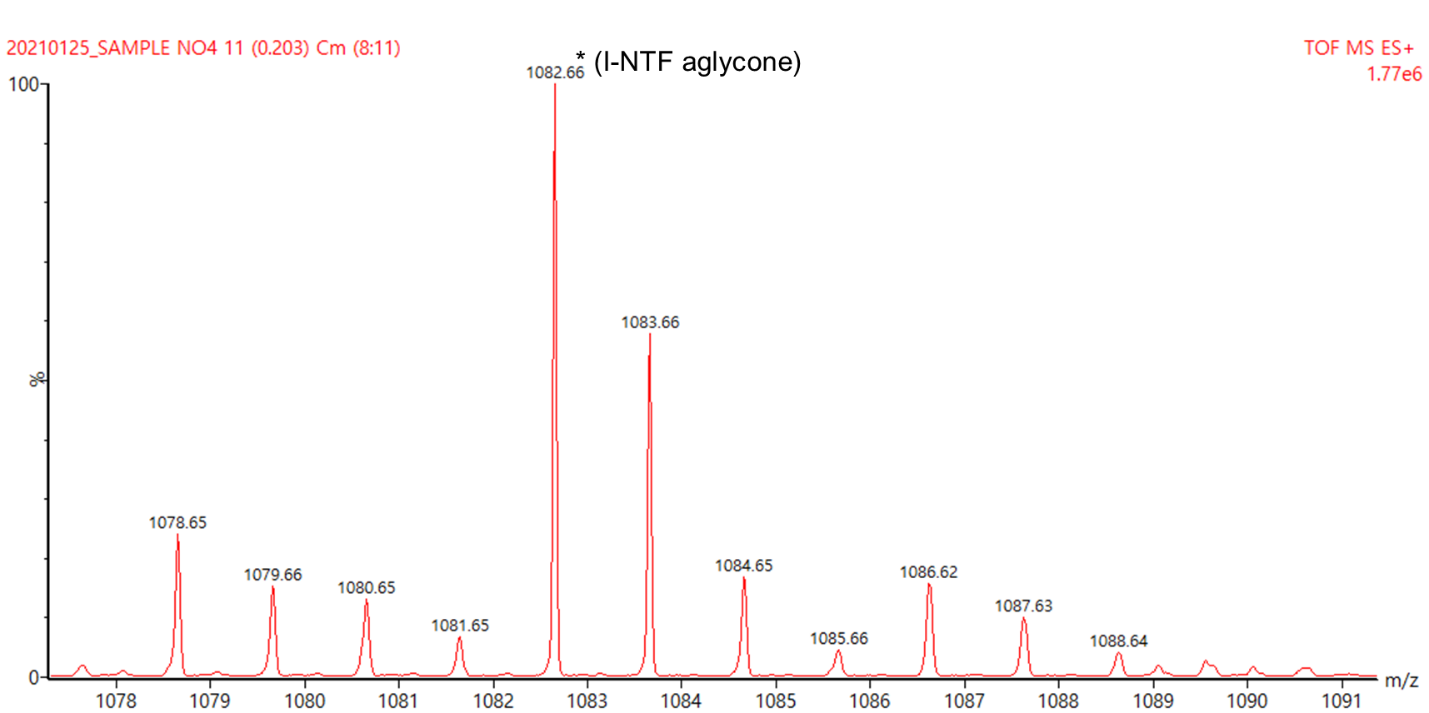


**Supplementary Figure S8.** Comparison of HPLC analysis results of I-NTF aglycone production yields after seven days of culture.


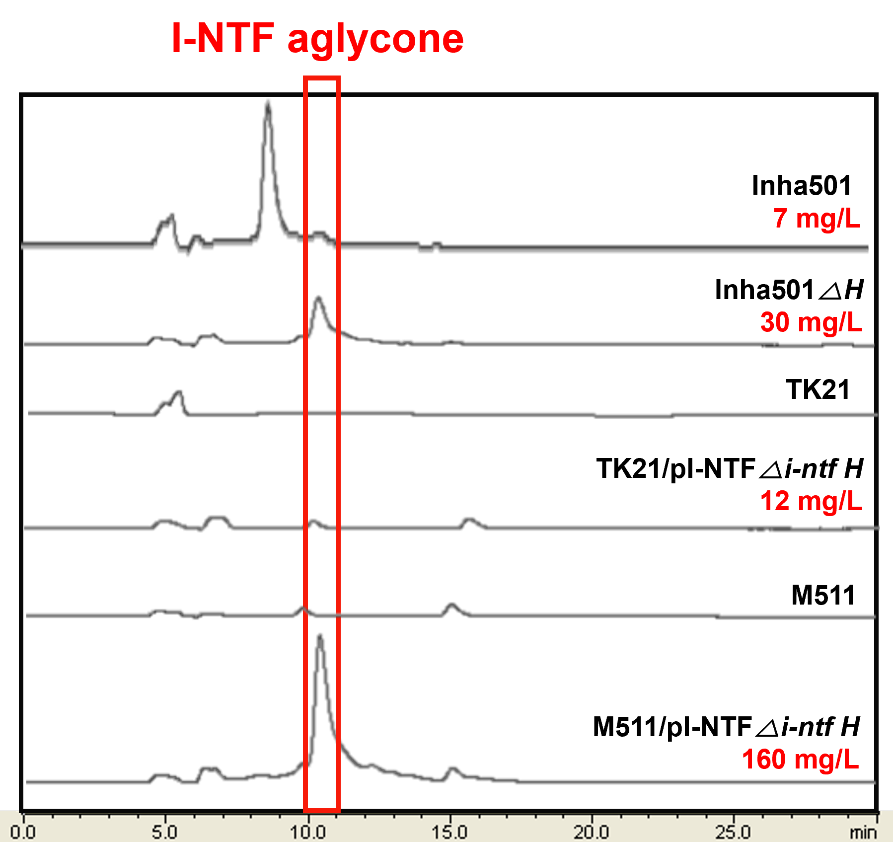


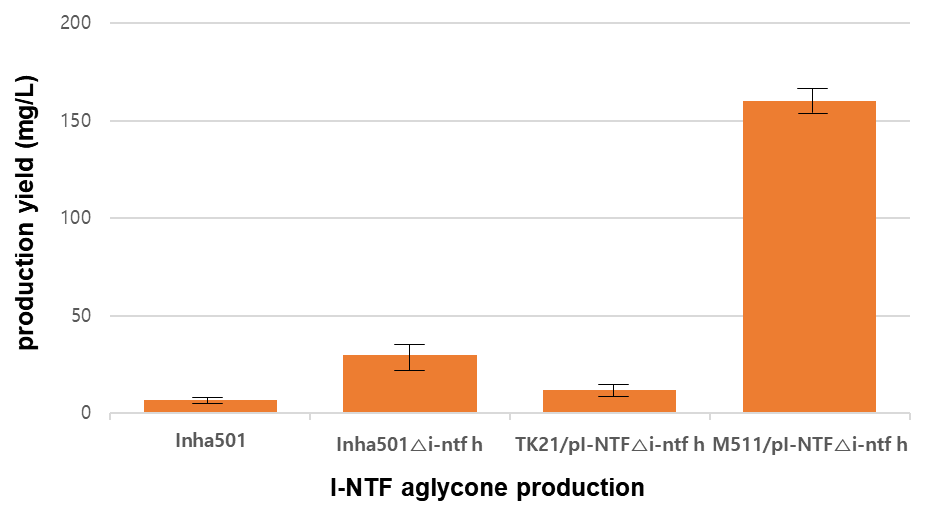


**Supplementary Figure S9.** Antifungal activity of I-NTF production strain and I-NTF aglycone production strain against *C. albicans* and 11 phytopathogenic fungi.


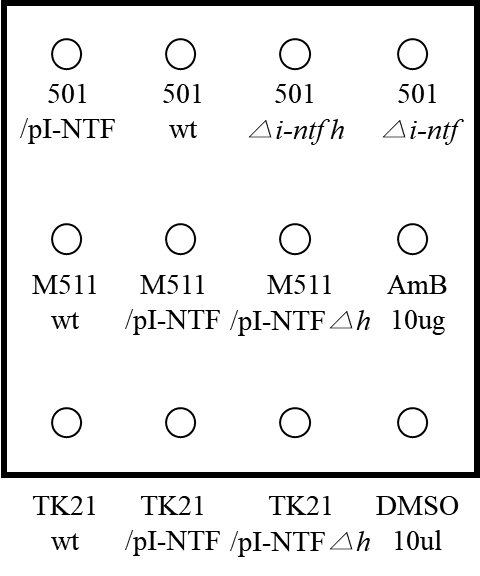


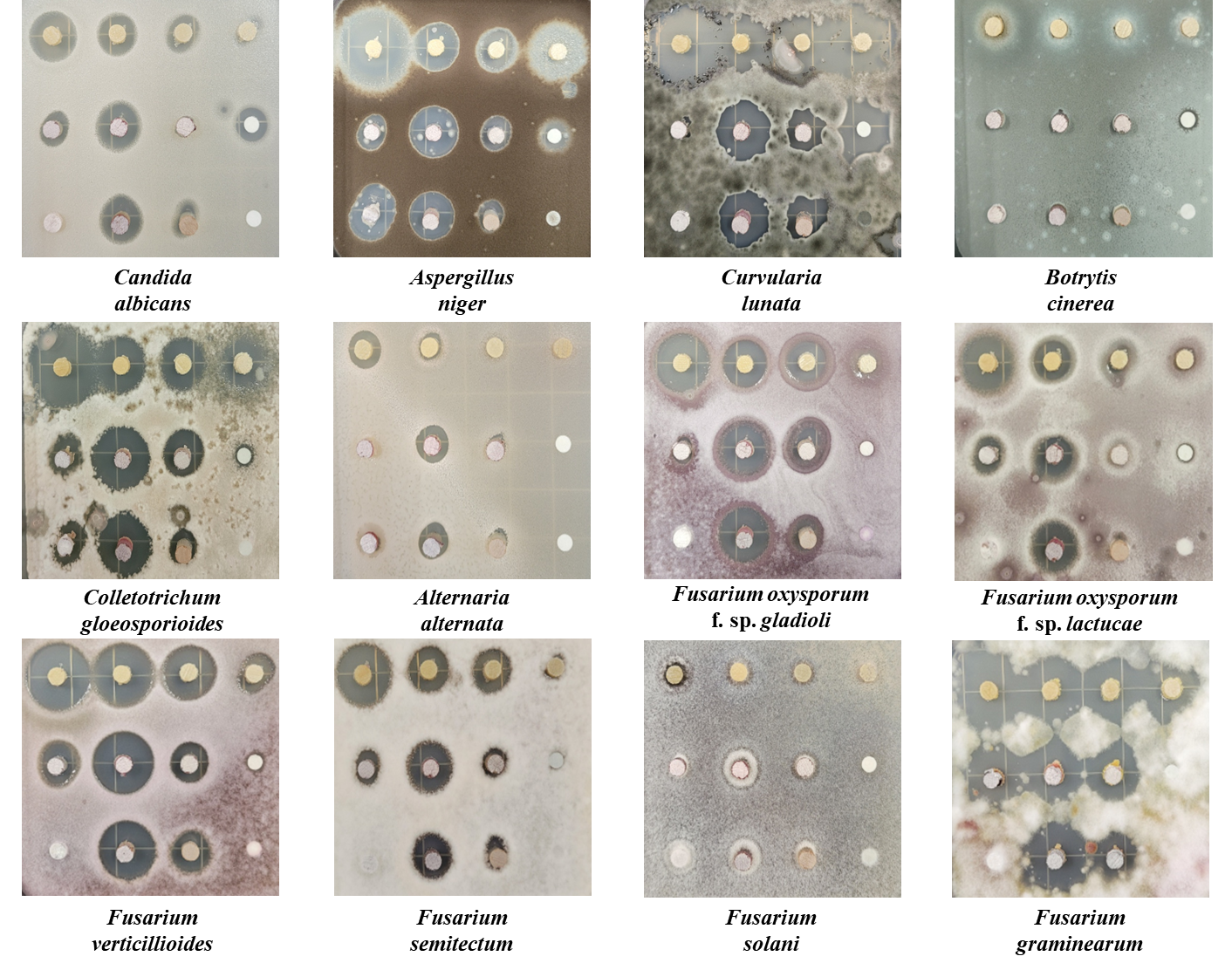
**Supplementary Figure S10.** *In vitro* antifungal activity of I-NTF and I-NTF aglycone using the RPMI-1640. The minimum inhibitory concentration (MIC) values were determined by measuring the minimum concentration that changed color to yellow.


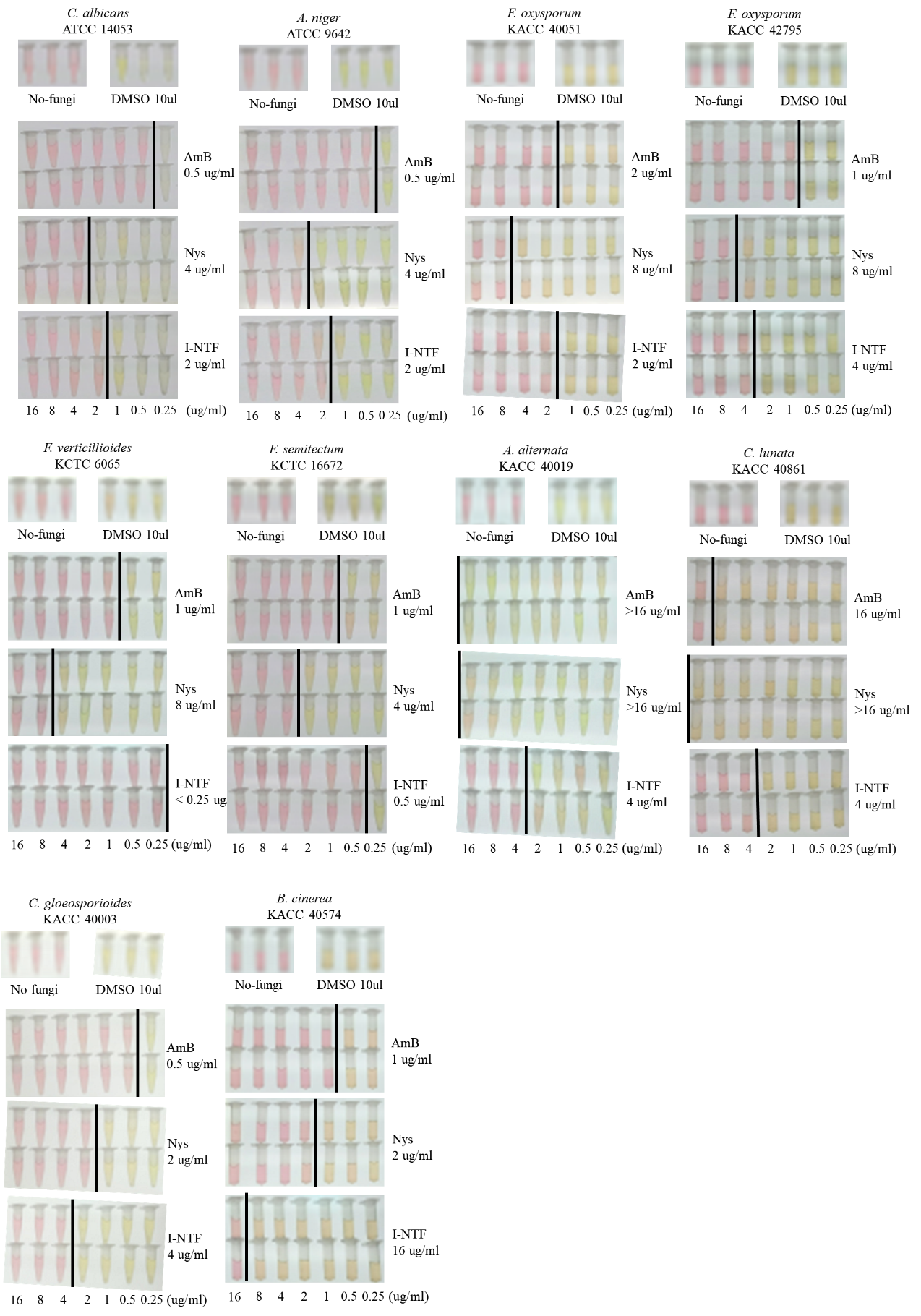


**Supplementary Table 1.** List of BGC check primers and qRT-PCR primers.

| **Primer** | **Primer sequence, 5’→3’** |
| --- | --- |
| 501-I-NTF up F | TGAGCCAGAGCCTCTGTCGA |
| 501-I-NTF up R | TCCTTGACGATACCGTCGTC |
| 501-I-NTF mid F | TGGCCCTGTTCGACCTGA |
| 501-I-NTF mid R | TCCTCCGGATAGCCGGAGA |
| 501-I-NTF dw F | ACAGCAGGTTCTCGAAGT |
| 501-I-NTF dw R | AGAAGCTCGACTACCTGA |
| I-NTF PKS I F | GCTTTCAAGGAGTTGGGCTT |
| I-NTF PKS I R | GGAGGACAGGACGAATTCCA |
| I-NTF PKS V F | CGGTCACGCTGATATTCGAC |
| I-NTF PKS V R | CGTTTCGTTCCACTTCGACA |
| I-NTF PKS IX F | CTGGACGTCGAGGGACTCTA |
| I-NTF PKS IX R | GACACCGGTCCAGGAGAAT |
| I-NTF B F | CTGCAGTTCCTGCTCTACCT |
| I-NTF B R | GTGATGTCGTGGCACTCG |
| I-NTF D F | AGGCTGATGATGCTGGTCA |
| I-NTF D R | ATCTCGCTGCTCGGCATAG |
| I-NTF E F | CCATCGTCATCGCACTGTTC |
| I-NTF E R | GATCCACAGCAGAGCGTATC |
| I-NTF H F | AACAGCTGGATACCTGCCAT |
| I-NTF H R | GCGTGTTGTTCGTCTCGTAT |
| I-NTF K F | TCGGAGTAGACCTTCAACCG |
| I-NTF K R | GGAGAAGCTCTCCAACACCT |
| I-NTF I F | TCGGAGTAGACCTTCAACCG |
| I-NTF I R | GGAGAAGCTCTCCAACACCT |
| I-NTF U F | GGAACATTTCTCCGGCCTTG |
| I-NTF U R | AACTCGACCTGTGCAACGTA |
| I-NTF W F | ACGCACTTCGGTGAGAAGTA |
| I-NTF W R | TGTCGACGGAGATGTAGAGC |
